# The Metropolitan Hospital Sunday Fund

**Published:** 1911-12-30

**Authors:** 


### THE METROPOLITAN HOSPITAL SUNDAY FUND.

### An Animated Debate at the Mansion House.

The annual meeting of constituents of this Fund was was held at the Mansion House on Thursday, December 21, 1911, under the Chairmanship of the Lord Mayor, the Right Hon. Sir Thomas Crosby, M.D.

The notice convening the meeting and letters of regret having been read from, amongst others, the Bishop of St. Albans, the Bishop of Islington, Prebendary Anderson, and the Rev. Simpson Davies, the minutes of the last annual meeting were read. The Rev. L. S. Lewis, alluding to the phrase used in the minutes "overwhelming majority," objected that, as a resolution in which he was concerned was almost carried, numbers of votes should be stated in the minutes in future. In answer to the Chairman as to whether he would wish a proper division to be taken, he said he did not wish that, but that if any indication of the degree of support were given in the minutes about one proposition, it should be given for all.

The Bishop of London moved

"That the Report of the Council for the year 1911 be, and is hereby, received." He said he did so with much pleasure. Of course, all would regret that the amount fell £3,000 behind that of the year before, but there may have been reason for that, and efforts must be made to recover the lost ground this year. He always looked upon this Fund as one of the great golden bridges between the rich and the poor in London. He had lived so much among the poor of London that he knew quite well what the hospitals were to the poor; he could not contemplate London without these glorious hospitals standing in the midst of the masses. The golden bridges enabled the funds to be sent for the benefit of the poor without any chance of doing harm to their poor friends. He looked upon such a fund as this as a great bond of union between all religious people of London and all men of good will. He loved to meet there, as he always did, men of other denominations than his own, and there was none whom he missed more than he did his old dear friend the Jewish Rabbi, one of the kindest-hearted and most generous of men. (Applause.)

Sir J. C. Bell seconded the resolution, remarking upon the large amount of work represented, and sharing in the regret at the reduction of the total amount received. Still, he hoped extra efforts would be made next year to recoup the amount. Much had been heard of charities falling short, but he hoped that would never be allowed to apply to this particular fund, for it was the very backbone and essence of protection of the poor of this country.

### The Case of St. George's Hospital.

Mr. Stow (St. John's, Notting Hill) wished to ask a question before the resolution was put, namely, why the Distribution Committee of the Fund had accepted the assurance of the authorities of St. George's Hospital that money paid to the Medical School from the funds of the hospital really went to the sick poor, when King Edward's Fund had not been satisfied, and had, in consequence, made no grant to that hospital?

Sir William Church, replying to the question, said the King's Fund had given no grant to St. George's Hospital this year for the very good reason that St. George's Hospital did not apply for one. On behalf of the Sunday Fund, he would say that they had accepted the word of the Chairman of St. George's Hospital, and no reason seemed to exist for doubting his word as an honourable man. (Hear, hear.) This Fund did not enter into the constitution and government of the hospitals in the same way that the King's Fund did. The letter ran: — "Dear Sir Edmund, in reference to the visit of the deputation yesterday from St. George's Hospital to your Distribution Committee, and the conversation that took place, I am glad of an opportunity of stating, in the clearest possible manner, that not one penny of the money subscribed by the Hospital Sunday Fund, or from any other source, to the general funds of this hospital is used for any purpose whatever save the maintenance and treatment of the patients. We could easily subsidise medical teaching in this hospital if we wished to out of the ample Discretionary Fund that we have at our disposal. I hope this may remove any misunderstanding on the part of those who last year raised this question at the annual meeting. Yours truly, A. E. West."

Mr. Stow said he was quite willing to accept that explanation given by Sir William Church, but in view of the facts which had been disclosed, and which he proposed to refer to, he thought this meeting would come to the conclusion that though Sir William Church's explanation was satisfactory in one sense, it would not be accepted as satisfactory to the large number of constituents of the Fund represented by the various congregations. He thought it was clear that had St. George's Hospital applied for a grant, that application would have been ignominiously refused. ("Why?")

The Chairman interposed to say that he certainly would not have refused such an application.

Mr. Stow said he would admit there would have been one exception. He drew attention to the first Law of the Constitution of the Fund: "The congregations which have forwarded contributions to the Fund during two successive years shall be entitled to a voice in the management of the Fund during the next year following the last of such contributions . . ," and said this was the one occasion when those who represented the contributors to such an important Fund had the right to speak on the matter. He then passed in review the history of the controversy concerning St. George's Hospital, and its alleged diversion of funds towards the support of its Medical School, and he quoted the relevant passages from the Report of Sir Edward Fry's Commission, including the following: "We venture to submit that the distinction between the hospital and the school should in every case be drawn, not only definitely and exactly, but with such clearness that it may be understood by the general public, and so that no question may arise as to the destination and application of moneys contributed, whether by the King's Fund or from any other source." Almost all the great London hospitals had accepted the finding of the Commission, and had been loyal to that Report, but great objection had been raised as to the position of St. George's Hospital, and last year the King's Fund Distribution Committee inquired into the whole matter in reference to that hospital. That Fund did not accept anybody's assurance, but they were in communication with St. George's Hospital, they visited the hospital, and inspected the clinical laboratory, the museum, and school buildings, and they subsequently made their report. In that report it was stated that the cost of laboratory investigations, etc., at St. George's Hospital worked out at about two-and-a-half times the average if St. George's Hospital was included, or slightly over two-and-three-quarter times if St. George's was excluded. Any fair man would wish to know why there was such a difference, and the conclusion was arrived at that the Fund ought to mark its disapproval of an expenditure so far in excess of the average, whether such excess arose from extravagance, from a contribution to the Medical School, or from the clinical laboratories being designed not only for hospital requirements, but also for purposes of research. They added that their own conclusion was that a larger proportion of the expenditure should have been borne by the school, and that the school should have paid to the Hospital in respect to the year 1909, and if necessary 1910, the difference between the £1,000 and the charge borne to the hospital in respect to specific work done for the hospital patients, which amounted in 1909 to £1,875. But the Sunday Fund had made no inquiry into St. George's Hospital, but the assurance of the St. George's Hospital authorities had been accepted. He did not say they were bound to follow the lead of the King's Hospital Fund, but one great Fund had found the finances of the hospital in question faulty. Was the Sunday Fund going to approve what the King's Fund rejected? This meeting was not large numerically, but it represented many thousands of pounds; and it was the one occasion in the year when the constituents had the right to express either approval or disapproval of the action of the Distribution Committee. He therefore moved, as an amendment, "That as King Edward's Hospital Fund, after careful investigation into the accounts of St. George's Hospital, has felt itself unable to make a grant to that hospital, the Hospital Sunday Fund refuses in such a matter to encourage what King Edward's Fund condemns, and accordingly adopts the report of their Council for 1911 with the exception of the grant of £1,554 9s. 8d. awarded to that hospital."

The Rev. L. S. LEWIS seconded.

The CHAIRMAN said such an amendment must have reference to the future, as the money in question had already been paid months ago.

Mr. STOW asked if constituents were to understand that when they attended to represent congregations they had no voice. They had only within the last week or so received notice of this meeting. He was not sure of being at the meeting next year, and he asked when he could feel an effectual voice could be raised.

Mr. F. H. NORMAN pointed out that the Laws of Constitution settled the matter, and the course now followed accorded with the usual custom. After the report had been approved by the Council the money was distributed. The desired alteration could be brought about by proposing to change the Constitution. He did not express an opinion as to whether Mr. Stow was right or wrong in his contention.

Mr. STOW retorted that an alteration of the Constitution was not his objective, but he asked for a ruling as to the present position. Either the constituents had a voice in the affairs of the Fund or they had not. If the former, his amendment was in order; if not, he did not know why the meeting had been held.

The CHAIRMAN replied that a remedy would be to elect a fresh Council next year. But he understood Mr. Stow's chief complaint to be against St. George's Hospital.

The Hon. SYDNEY HOLLAND asked whether the answer to Mr. Stow was not that the subscribers to the Fund had passed a Constitution of the Fund—for good or for bad—and that the money had been distributed according to the Constitution, and, therefore, according to the views of the Distribution Committee. Except for one or two critics, the Fund had hitherto been administered without hostile comment.

Mr. WILLIAMSON said he could understand that any ruling must be prospective, but he dissented from the assumption that criticism was necessarily from a hostile motive; as one who was accustomed to meetings in the City, he protested against such an idea. All present had one object: the furtherance of the benefit of the hospitals, especially under the auspices of the Sunday Fund, and he did not see that there was any need to alter the Constitution of the Fund. So long as criticism was kept within reasonable limits, he believed it would do nothing but good, and that it would also increase the popularity of the Fund. In reference to the regret expressed by the Bishop of London that the Fund was even £3,000 less than last year, he believed the Sunday cinematograph shows had had an adverse effect upon the Fund. He also deprecated the tone of the Hon. Sydney Holland's letter on the subject in the *Westminster Gazette*.

The Hon. SYDNEY HOLLAND reminded the speaker that the object of the meeting was not to discuss a letter he had written to a newspaper; and that the letter was better than some of his opponents deserved.

The Rev. L. S. LEWIS asked the Chairman to accept the following amendment: "That as King Edward's Hospital Fund, after careful investigation of the accounts of St. George's Hospital, has felt itself unable to make a grant to that hospital, the Hospital Sunday Fund refuses in such a matter to encourage what King Edward's Fund condemns, and wishes it to be an instruction to the distribution committee for next year to fall in line with King Edward's Fund on this matter."

Mr. STOW, after some discussion as to procedure, seconded the foregoing amendment proposed by Mr. Lewis.

The Rev. L. S. LEWIS, speaking to his amendment, said Sir William Church, by a slip of the tongue, said St. George's Hospital had not applied for the grant; but that was not quite the position: they did apply for the grant, but withdrew that application. All present would appreciate the difference between dismissing a person from one's service and permitting that person to resign. That was what happened in respect of St. George's Hospital. Mr. Lewis proceeded to support his position by quoting from the *Times* report of December 12 of a meeting of the King's Fund at St. James's Palace, when the Speaker of the House of Commons presided. The Bishop of London had spoken about the golden bridge between the rich and poor of London formed by the hospitals. He (the speaker) yielded to no one in interest in them, and it was because they were such splendid institutions that those interested were bound to see that everything was right. The man who adulterated bread was worse than he who adulterated wine; and the hospitals were the equivalent of the bread to the sick poor. He wanted to ensure that none of the gold, in its passage over the bridge, should pass through the chinks. The quarrel with St. George's was entirely the result of outside pressure, and in consequence of that the Commission which had been alluded to was appointed, and consisted of trained minds. They found the criticisms were justified, and that grants which had been passed from the hospital to the school should be looked upon as a debt, as something refundable. That became a dead letter. But once again pressure was brought to bear, and this time by a great federation of working men; and as a result of that, last year the King's Fund only made a conditional grant, as they found the hospital was not behaving in accordance with the finding of Mr. Justice Fry's Commisson. But last year's meeting made it clear that the annual meeting of constituents was largely a farce, for the money had already been voted. And the position was that while from one great fund the hospital had not got its grant because it had not satisfied the Fund that its behaviour had been right, that hospital could snap its fingers at the other fund because it had accepted the assurance of a deputation, supported afterwards by an assurance in writing by an official.

A Constituent thought it important before a vote was taken that the meeting should grasp the fact that the Fund had appointed a Distribution Committee to administer the Fund, and that function had been done in a way which gave general satisfaction. The question now before the meeting arose upon a state of affairs disclosed by a Commission appointed by the King's Hospital Fund. That Fund, having its own rules and regulations, considered they would not make a grant to the hospital in question. This Sunday Fund, having its own regulations and feeling confidence in its Distribution Committee, which in turn had confidence in the Chairman of St. George's Hospital, felt prepared to make a grant. Therefore he thought the present meeting should hesitate long before passing a vote of censure on its own Distribution Committee. (Applause.) But the Committee might be asked to look more closely into the administration of the funds at St. George's Hospital before the next distribution.

Sir William Church said a good many different issues had been mooted in that hall on that occasion, and he believed he could put some of them straight. The Distribution Committee had always endeavoured to carry out to the best of its ability the wishes of the Council of the Fund. It would be remembered that the same charge was made against the Distribution Committee last year, that they made a grant to St. George's Hospital, that Hospital having used part of its money for the benefit of the medical school attached to it. But before the Sunday Fund Distribution Committee made any grant to that Hospital this year and last year they not only had the word of the Treasurer and Chairman of the Hospital that no part of the grant made by the Sunday Fund would be used for any other purpose than for the benefit of the patients at St. George's Hospital, but they also looked into the accounts of the Hospital and saw that it had a discretionary power over much larger funds than the grant which the Fund made. The St. George's Discretionary Fund was one in which the subscribers to the Hospital indicated their wish that the Hospital authorities should use the money as they thought best. Those who subscribed the money under that head did not make it a *sine qua non* that that money should be used wholly in the interests of the patients. It was not for this present meeting to go into the differences which existed between the government of St. George's Hospital and the King's Fund. It had been said that he, Sir William, was not correct in saying that St. George's made no application this year to the Fund; but he thought he really was correct. What happened was, that St. George's refused to conform to the requirements of the King's Fund for the preceding year, and by that omission they became ineligible for a grant, according to what was the custom, that if a grant was made to a hospital and it was not placed to its right use, that hospital put itself out of the running for the following year. In the case of the King's Fund, in the grant of money for 1909, St. George's refused to pay any sum whatever to the School. He was not there as representing St. George's Hospital in the least, but he believed they did it from very honourable principles. They said, whether it was thought to be right or not, that the money had been spent in supporting the laboratories which were necessary for the use of the patients. They said their word might be doubted, but the accounts were open to inspection. The action of the King's Fund had been unnecessarily imported into this meeting; this Distribution Committee were only acting upon what he understood was the desire of the Council, that before giving a grant to any hospital to which a medical school was attached, the word of the Chairman of that Hospital should be obtained that no portion of the grant made by the Sunday Fund should be used for any purpose except for the use of its patients. They also took the precaution to see whether a hospital which had a medical school attached and might be found to have used any of the Sunday Fund money for the school, had a discretionary power over any money at all.

The Hon. Sydney Holland suggested that the meeting should be content with its own Distribution Committee, without going to the decisions of another Fund. As a much interested party in one hospital it was to his advantage that the grant should not be given to St. George's, but he considered the Fund had a good Distribution Committee, and they had given much satisfaction, except to one or two people.

The Vicar of Whitechapel (the Rev. G. M. Hanks) said it was well known that the work of such a Fund as this was dependent largely on the public confidence, and they were there to try and restore that confidence. The rift in that confidence could not be healed easily, but it could be healed to-day. It would be very serious for the Fund if the present discussion were carried to the vote, even if the Distribution Committee had a considerable majority, and therefore he hoped it would be possible to get this amendment withdrawn. Assuming that confidence was broken, there was a strong feeling among working men of London that it was not the business of this Fund to help rich gentlemen to educate their sons to a profession, and they were exceedingly jealous that funds given for the sick poor should not go to such a purpose. It was true that St. George's Hospital had taken in this matter an exceedingly noble stand; they had declined any longer to sail under the commonplace of a Discretionary Fund, and he honoured the Hospital for that; their withdrawal from application for a share was a very strong and independent action. But how did it affect the confidence of the City? If there were two charities in a neighbourhood, and the administrators of one saw fit to attach certain conditions to grants, and there was another fund where no such condition obtained, then the applicants could snap their fingers at the conditions of the first. In a greater walk of life, that was what was happening in this connection. Admittedly St. George's Hospital had not applied to the King's Fund because it was not prepared to abide by the conditions, yet they came to the Sunday Fund and got it. He would be glad to see St. George's Hospital have the grant, because it was doing a magnificent work. He was most anxious that the confidence of the public should be restored in the Fund. When, last year, he was told that the distribution had been made, and it could not be undone, he was satisfied when he understood that very strict inquiry would be made before another grant was made. But he did not now see evidence that the undertaking which brought peace at the similar meeting last year had been kept. There was a chance of peace to-day, and he urged the meeting to take it.

The Bishop of London said he had sympathy with two things: (1) that there should be free scope for the expression of any grievance felt in regard to such a Fund as this, (2) that no money which was subscribed for one object should be spent upon another. But where he differed from those two priests of his from poor districts was that for him the word of honour of the Chairman of a great hospital, engaged in a labour of love was sufficient; Mr. West's word was, to his Lordship, as good as his bond. If he had not that feeling, he would have supported those faithful priests of his. But he hoped that the assurance of Mr. West, which had satisfied him, would satisfy others also.

The Rev. L. S. Lewis asked if the Lord Mayor would give his personal assurance that this matter should be most thoroughly thrashed out before the next meeting of the constituents of the Fund. This his Lordship said he would do, and Mr. Lewis thereupon, with the consent of his seconder, Mr. Stow, withdrew his amendment, and the original resolution was carried unanimously.

The Council for the year 1911 was re-elected for 1912, with the addition of the Ven. Archdeacon of London, the Rev. Prebendary Perry, M.A., the Rev. J. Allen Bell, the Rev. H. A. Hall, the Rev. Simpson Johnson, the Rt. Hon. Sir Thomas Vezey Strong, and Messrs. Arthur F. Charrington, and Andrew Motion.

June 9 was fixed as the date for Hospital Sunday, 1912.

A Strong and Tactful Lord Mayor.

With perfect unanimity, on the motion of Mr. F. A. Bevan, seconded by the Rev. E. P. Hilliard, the Lord Mayor was cordially thanked for his occupancy of the chair. Mr. Bevan said the function of that afternoon had been no sinecure, and the manner in which the duty had been carried out proved that the Lord Mayor was the right man in the right place. He cordially wished him health and strength to carry out his various duties.

The Lord Mayor, in acknowledging the compliment, said he had listened with great pleasure to the discussion which had taken place. Such discussions were always fraught with some good, and this one had been carried out ably and generously. If during the coming year any of those present would consent to look at the question of the hospitals, he would lend them a pair of medical spectacles; then he was sure they would come next year with a different view of hospitals and their needs and the relationship of the medical schools to them.

